# Changes in the rumen development, rumen fermentation, and rumen microbiota community in weaned calves during steviol glycosides treatment

**DOI:** 10.3389/fmicb.2024.1395665

**Published:** 2024-06-24

**Authors:** Kexin Wang, Maocheng Jiang, Yuhang Chen, Yuncheng Huang, Zhiqiang Cheng, Osmond Datsomor, Shakib Mohamed Jama, Liping Zhu, Yajing Li, Guoqi Zhao, Miao Lin

**Affiliations:** ^1^Institute of Animal Culture Collection and Application, College of Animal Science and Technology, Yangzhou University, Yangzhou, China; ^2^Zhucheng Haotian Pharm Co., Ltd., Zhucheng, China; ^3^Institutes of Agricultural Science and Technology Development, Yangzhou University, Yangzhou, China; ^4^Joint International Research Laboratory of Agriculture and Agri-Product Safety of Ministry of Education, Yangzhou University, Yangzhou, China

**Keywords:** steviol glycosides, rumen, development, fermentation, microbiota

## Abstract

Early weaning leads to weaning stress in calves, which hinders healthy growth and development. As an excellent sweetener applied in food, steviol glycosides (STE) has also been shown to exhibit positive biological activity in monogastric animals. Therefore, this study aimed to evaluate the impact of incorporating STE as a dietary supplement on rumen development, fermentation, and microbiota of rumen in weaned calves. This study selected 24 healthy Holstein bull calves and randomly allocated them into two groups (CON and STE). The results indicated that supplementation STE group improved rumen development in weaned calves, as demonstrated by a marked increase in the weight of the rumen, as well as the length and surface area of the rumen papilla. Compared with the CON group, the concentrations of total volatile fatty acids (TVFA), propionate, butyrate, and valerate were higher in the STE group. Moreover, STE treatment increased the relative abundance of *Firmicutes* and *Actinobacteria* at the phylum level. At the genus level, the STE group showed a significantly increased relative abundance of *Succiniclasticum*, *Lachnospiraceae_NK3A20_group*, and *Olsenella*, and a decreased relative abundance of *Acinetobacter* compared to the CON group. *Pusillimonas*, *Lachnospiraceae_NK3A20_group*, *Olsenella,* and *Succiniclasticum* were significantly enriched in rumen chyme after supplementation with STE, as demonstrated by LEfSe analysis. Overall, our findings revealed that rumen bacterial communities altered in response to the dietary supplementation with STE, and some bacterial taxa in these communities may have positive effects on rumen development during this period.

## Graphical Abstract

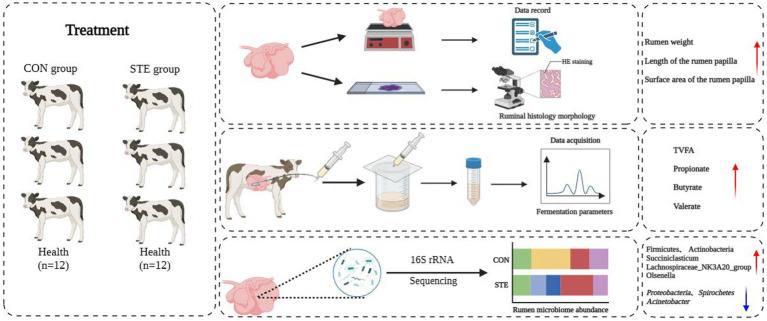

## 1 Introduction

With improved living quality, people are paying more attention to good taste while ensuring food provides nutrition. The balanced nutrition and taste requirements of ruminants for food are similar to humans’ dietary requirements. As described by the Pavlovian reaction, the secretion of digestive juices is influenced by appetite, and the digestion and absorption of feed are also affected (Tellez et al., 2016). Sweeteners play a crucial role in enhancing the feeding efficiency of ruminant animals due to their unique taste stimulation (Neumann et al., 2005). The sweetness of these additives stimulates the animals’ taste buds, transmitting signals to the cerebral cortex and the digestive system (Gaillard et al., 2017; Iwamura et al., 2020). This stimulation increases saliva, intestinal fluid, pancreatic fluid, and bile secretion in the digestive tract (Lee et al., 2021). The peristalsis and digestive enzyme secretion in the gastrointestinal tract are enhanced, promoting feed digestion and nutrient absorption (Ma J. et al., 2020). Previous research has shown that adding essence and spices to ruminant diets can enhance taste and smell, increasing food intake (Hussein et al., 2018). Furthermore, supplementation of sweeteners in the diet has been shown to increase the average daily gain of cattle (McMeniman et al., 2006; Ponce et al., 2014). Sweeteners contribute to animal growth, development, production performance, and feed utilization efficiency.

Steviol glycosides (STE) is an emerging natural sweetener derived from the herbal plant stevia (Chatsudthipong and Muanprasat, 2009; Lemus-Mondaca et al., 2012). It is a zero-calorie sweetener 470 times sweeter than sucrose (Salehi et al., 2019). STE has been discovered to possess immune regulatory activities and positive effects regarding anti-inflammatory, antioxidant, and antibacterial properties and maintaining blood lipid levels (Pariwat et al., 2008; Casas-Grajales et al., 2019; Wei et al., 2021). Moreover, scientific studies have demonstrated that STE also exhibits antibacterial properties in monogastric animals such as chickens and rats (Lopez-Carbon et al., 2019; Alavala et al., 2020; Jiang et al., 2021). As the biological activity of STE has been discovered, there is growing interest in their application in animal production. STE has demonstrated potential effects as a bioactive substance in goat digestion rate and rumen fermentation in animal experiment (Xu et al., 2021). Additionally, dietary STE supplementation significantly increased *Bifidobacteria* and decreased *Escherichia coli* of the cecal digesta in broilers (Wu et al., 2019). Recent research has investigated that the total or partial replacement of sugar with STE influences the general state of health, especially the human microbiota’s response. This study demonstrated the modulatory role of STE on the human microbiota and on the fermentation processes that determine the essential SCFA synthesis in maintaining homeostasis (Gatea et al., 2021). These results indicate that STE may play an important role in regulating the rumen microbial communities. To our knowledge, no studies have reported the impact of STE on rumen microbial communities in calves.

Calves are crucial to the success and sustainability of dairy farms as they are the backup force and the future hope for high milk production. Proper management of calves feeding is essential for the profitability of the dairy industry. The rumen is a vital organ in ruminants due to its connection with nutrient metabolism, uptake, and transport (Nishihara et al., 2019). During this process, the development of the rumen in calves is extremely important for their growth and development, and appropriate feed intake plays a crucial role in its complete development. Reducing production costs and accelerating rumen development in calves due to early weaning is currently a common feeding method for calves (Ma J. et al., 2020). However, early weaning will lead to weaning stress. During this stage of development, the calves’ immune organs and digestive system are still developing, making them more susceptible to gastrointestinal issues and threatening their health (Browne et al., 2021). The microbiota is involved in regulating the development and health of the gastrointestinal tract. Its importance lies in providing essential metabolites, maintaining metabolic function, supporting immune system development, and defending against pathogens, which are crucial for overall host well-being (Guo et al., 2020). In recent years, an increasing emphasis has been on investigating the relationship between host performance and microbial composition (Cunha et al., 2019; Sofyan et al., 2019), the relationships between gastrointestinal flora and milk production in dairy cows and calf diarrhea (Sofyan et al., 2019; Ma T. et al., 2020). Some scholars have demonstrated that dietary STE supplementation affects the gut microbiota of chickens (Wu et al., 2019; Jiang et al., 2021). Additionally, previous studies have indicated that early intervention of starter feed contributes to the prompt colonization of rumen microflora, leading to high levels of volatile fatty acids (VFA) in the rumen, which may stimulate the early development of rumen epithelium tissue (Lin et al., 2019). It is unclear whether dietary STE affects calf rumen development, fermentation parameters, and microbiota. Therefore, this study aimed to determine the effects of dietary supplementation with STE on rumen development and microbiota in weaned calves.

## 2 Materials and methods

The experiment was conducted at the experimental farm of Yangzhou University (Yangzhou, China), and the Yangzhou University Institution Animal Care and Use Committee (IACUC) approved all procedures involving animals (SYXK (Su) 2021-0026).

### 2.1 Animal experimental design

The experiment was conducted in the experimental base of the Animal Nutrition and Feed Engineering Technology Research Center of Yangzhou University in Jiangsu Province, China. In this study, twenty-four Holstein bull calves (60-day-old, body weight 72.25 ± 1.61 kg) were used and marked with ear tags. Calves in this study were randomly assigned to two dietary treatment groups, taking into consideration their initial body weight: CON with no STE (CON group, *n* = 12) and STE (STE group, STE accounted for 0.2% of the basal diet, *n* = 12). STE was supplied by Zhucheng Haotian Pharm Co., Ltd. (Total glycosides: ≥ 87.5%; main ingredients: rebaudioside A ≥39.9%, stevioside ≥30.4%; lot: M20220316, Zhucheng, Shandong, China). STE supplementation was delivered as granules mixed with the starter diet. Calves in the 2 groups were housed in 24 pens, with 1 calf in each pen. All calves received regular feedings three times a day at fixed times: 07:00, 14:00, and 21:00. During the experiment, calves freely consumed starter feed, oat hay, and clean drinking water. The composition and nutrient levels of starter feed are detailed in Table 1. The one-week adjustment period was followed by 8 weeks of the experimental period.

**Table 1:** Ingredients and nutrient levels of the experiment diet (%, dry matter basis).

Ingredient	Content	Nutrient levels	Content
Corn	52	DM	88.13
Soybean meal	25.5	Ash	6.34
Wheat bran	3.6	Crude protein	19.72
Barley	7.8	Crude fat	3.38
Vinasse	5.5	NDF^b^	12.75
Limestone	3.5	ADF^c^	6.68
CaHPO_4_	0.7	Ca	0.96
NaCl	0.8	P	0.84
Premix^a^	0.6	ME^d^, MJ/kg	2.77
Total	100		

a The premix provided the following per kilogram of the diet: VA 12000 IU, VD 5000 IU, VE 70 IU, Fe 100 mg, Cu 10 mg, Mn 30 mg, Zn 90 mg, Se 0.3 mg, I 1.0 mg, Co 0.2 mg.

b NDF, neutral detergent fiber.

c ADF, acid detergent fiber.

d Metabolizable energy, calculated from National Research Council (2001).

### 2.2 Sample collection

All rumen digesta samples were collected using a rumen fluid collector before slaughter, transferred into liquid nitrogen, and stored at −80°C. Meanwhile, the pH value of rumen digesta samples was measured. Six weaned bull calves in each group were slaughtered at 57 days of the trial period. After being fasted for about 12 h, calves were subjected to humane slaughter, bleeding, skinning, and evisceration. Subsequently, to prevent the contents from mixing between the gastrointestinal tract, the digestive tract was separated by a 2 mm nylon thread to clamp the digestive contents. Rumen tissue samples were immediately collected into a frozen tube after washing with cold phosphate buffer saline. Further, the other part of the rumen tissue was taken and put into 4% paraformaldehyde for histomorphology determination.

### 2.3 Stomachs weight and morphological changes

The digesta were removed from the stomachs of each weaned calves immediately after slaughter to determine the tissue weight of the empty rumen, reticulum, omasum, and abomasum by the electronic scale. Subsequently, the rumen tissue was rinsed with sterile phosphate buffer, and the tissue slices were prepared according to the methodology by Aguirre et al. (2014) as previously reported. A piece of ventral rumen specimen measuring approximately 2 cm × 2 cm was excised and immersed in a solution of 4% paraformaldehyde, wrapped in paraffin wax after dehydration. Then, the film was sectioned and stained with hematoxylin and eosin for morphological observation under a fluorescence microscope (FluoView FV1200, Olympus, Tokyo, Japan). A section image of the rumen papilla was taken. A quantitative morphometric analysis was performed to determine the length and width of the rumen papilla. In contrast, the surface area of the rumen papilla was calculated by multiplying the papilla length by the papilla width according to Ma J. et al. (2020). For morphological analysis of rumen papilla, a microscope equipped with Image-Pro Plus 6.0 software (Media Cybernetics Inc., Bethesda, MD, United States) was connected to a digital camera to record 2 slides per sample and at least 9 papillae per slice measurements. The average of all measured values was used for statistical analysis.

### 2.4 Determination of ruminal fermentation parameters

After the rumen fluid collection, a portable pH meter (Mettler Toledo AG Analytical, Schwerzenbach, Switzerland) was immediately utilized to detect the pH of rumen digestion. A colorimetric assay was used to detect the concentration of ammonia nitrogen in rumen digesta, according to a previous report (Broderick and Kang, 1980). The sample was prepared to measure the concentration of VFA in rumen chyme with minor modifications according to a previous description by Dai et al. (2017). In detail, the sample was thawed at room temperature and thoroughly mixed. Then, 2–3 mL of the rumen liquid sample was centrifuged at 12,000 rpm at 4°C for 10 min. Subsequently, 1 mL of the supernatant was aspirated and placed in a 1.5 mL Eppendorf tube. The supernatant was mixed with 200 μL 20% metaphosphate and was refrigerated at −20°C overnight. The following day, the samples were centrifugated at 12,000 rpm at 4°C for 10 min and filtered by a 0.22 μm filter membrane. After that, the resulting supernatant was carefully transferred to a new tube, and 1 μL was taken as the sample size. For the determination of VFA content, gas chromatography (GC9800, Shanghai Kechuang Chromatographic Instrument Co., Ltd., Shanghai, China) with capillary columns FFAP 123-3232 30 m × 0.32 mm × 0.25 μm (Agilent Technologies, Stevens Creek Blvd, Santa Clara, CA, United States) was used. The conditions were listed below: Nitrogen was used as a carrier gas, the injection hole and gasification chamber temperature were 220°C, and the flame ionization detector temperature was 200°C. The initial and final temperature of the column was 100°C and 136°C, respectively, with a heating rate of 3°C/min, and the column pressure was 0.15 Mpa. Furthermore, the proportions of ruminal VFA were calculated.

### 2.5 16S rRNA gene sequencing analysis of microorganisms in ruminal digesta

The first step was to extract and purify DNA from rumen digesta. Amplification of the V3–V4 region of the 16S rRNA gene was subsequently performed using 16S rRNA universal gene primes 341F (5′-CCTAYGGGRBGCASCAG-3′) and 806R (5′-GGACTACHV GGGTATCTAAT-3′) as described previously (Xie et al., 2021). The amplicon obtained from the 2% agarose gel was purified using the AxyPrep DNA gel extraction kit (Axygen Biosciences, Union City, CA, United States), following the instructions provided by the manufacturer. ABI StepOnePlus Real-Time PCR System (Life Technologies Foster City, United States) was utilized for quantification. Sequencing libraries of purified PCR products were constructed using an Illumina TruSeq DNA sample preparation kit (Illumina, San Diego, CA). Finally, the libraries were sequenced on the Illumina MiSeq platform (Buonocore et al., 2017). The UPARSE software (Uparse v7.0.1001, http://drive5.com/uparse/) was employed to cluster valid labels into operational taxonomic units with a similarity threshold ≥97%. The marker sequence with the highest abundance within each cluster was chosen as the representative sequence, and the RDP classifier (version 2.2) was used for biological classification utilizing the SILVA (SILVA.123.1_SSURef_Nr99) database. A Venn diagram was employed to represent the distribution of unique and common operational taxonomic units (OTUs) in different samples, and the saturation of sequencing was presented by Coverage. The two groups’ richness and diversity of rumen microbial communities were analyzed using the α diversity index (Sobs, Chao1, ACE, Simpson, and Shannon). Principal coordinate analysis (PCoA) and non-metric multidimensional scale (NMDS) based on weighted UniFrac distance were conducted to assess the dissimilarity between the rumen bacterial communities of the two groups of calves. Linear discriminant analysis (LDA) effect size (LEfSe) with LDA score >4 was used to determine specific bacteria that differed significantly between the rumen digesta of the two groups. Finally, to observe the correlation between the relative abundance of a specific bacterial group at the genus level and the concentration of VFA in rumen contents, Spearman correlation analysis was conducted.

### 2.6 Statistical analysis

IBM SPSS Statistics 25 (SPSS Inc., Chicago, IL, United States) software was used to perform an independent sample *t*-test to evaluate the significance of differences in the weight of the stomachs, rumen papillary morphology, and rumen fermentation parameters between the two groups. Welch’s test was then utilized to examine the α diversity of rumen microorganisms and the relative abundance of bacteria at both the phylum and genus levels. The β diversity of PCoA and NMDS between the two groups was calculated based on weighted UniFrac distance. All data are represented as mean ± standard error, and *p* < 0.05 was noted as a significant difference, 0.05 ≤ *p* < 0.10 was declared a trend. The Spearman correlation coefficient was computed to assess the correlation between rumen microorganisms and fermentation parameters. A significance level of *p* < 0.05 indicated a statistically significant correlation. A heatmap was generated to visually represent the correlations, where asterisks were used to indicate significant correlations.

## 3 Results

### 3.1 Effects of STE on the stomachs weight of weaned calves

**Figure 1:**
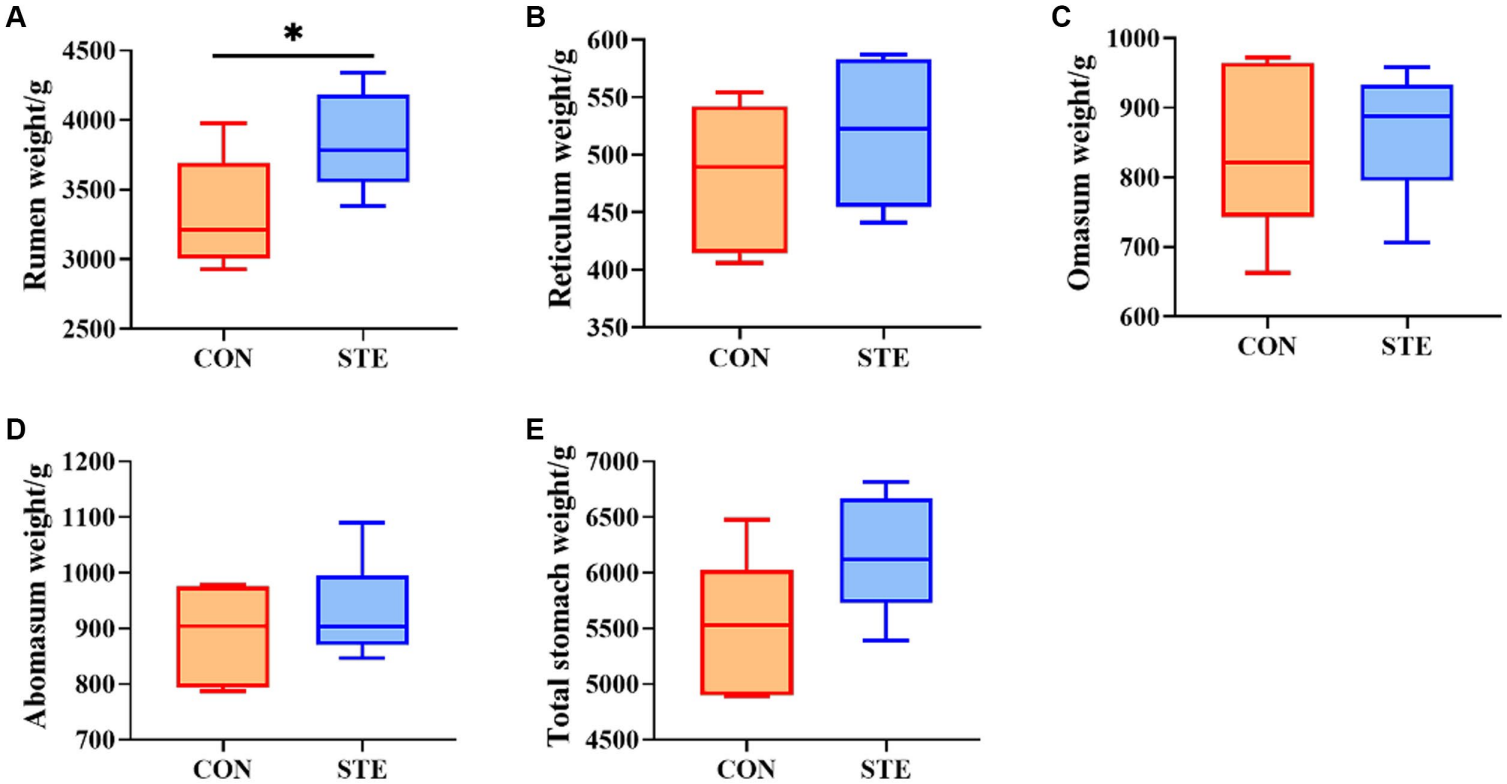
Effects of STE on stomachs weight. The weights of **(A)** rumen, **(B)** reticulum, **(C)** omasum, **(D)** abomasum, **(E)** total stomach in the CON and STE groups. Data were presented as the minimum to maximum (*n* = 6 per group). ^*^p < 0.05 compared to the CON group.

The effect of STE on the stomachs weight of weaned calves is shown in Figure 1. No significant differences were observed in the weight of the reticulum (*p* = 0.317), omasum (*p* = 0.631), abomasum (*p* = 0.442), and total stomach (*p* = 0.086) between the CON and STE groups. Notably, the weight of the rumen (*p* = 0.039) in the STE group was higher than in the CON group. Additionally, calves supplemented with STE presented an increasing trend in the weight of the total stomach (*p* = 0.086), with an increase of 11.17% compared to the CON group (CON vs. STE, 5533.00 g vs. 6151.17 g).

### 3.2 Effects of STE on ruminal histology morphology of weaned calves

**Figure 2:**
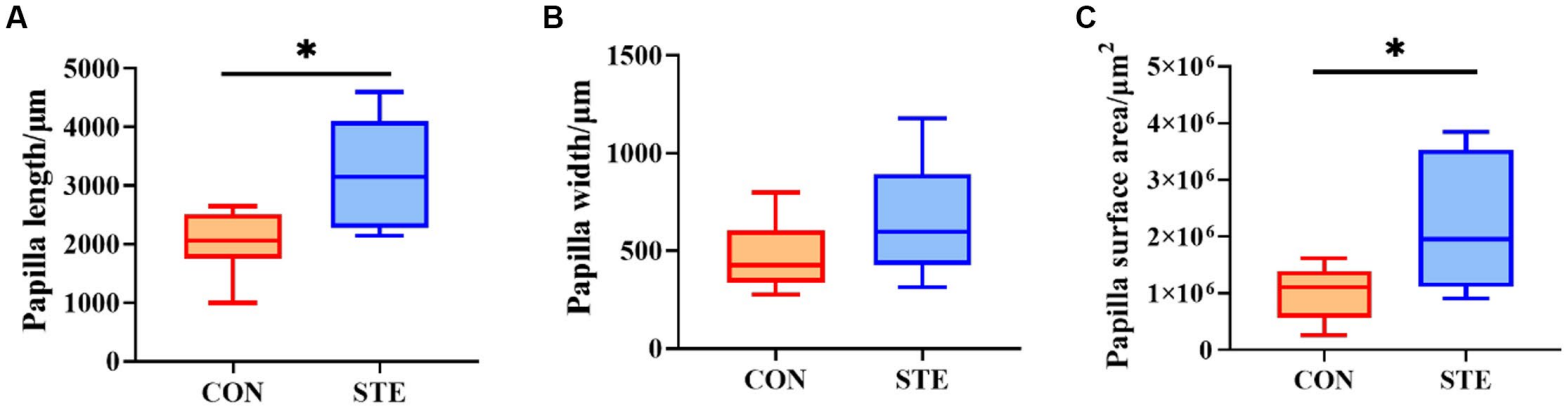
Effects of STE on ruminal histology morphology. **(A)** Papilla length. **(B)** Papilla width. **(C)** Papilla surface area. Data were presented as the minimum to maximum (*n* = 6 per group). ^*^p < 0.05 compared to the CON group.

Compared with the CON group, the length and surface area of the rumen papilla in the STE group were significantly increased (*p* < 0.05). However, the evaluation of papilla width indicated no significant difference between the CON and STE groups (Figure 2, *p* > 0.05).

### 3.3 Effects of STE on ruminal fermentation parameters of weaned calves

**Figure 3:**
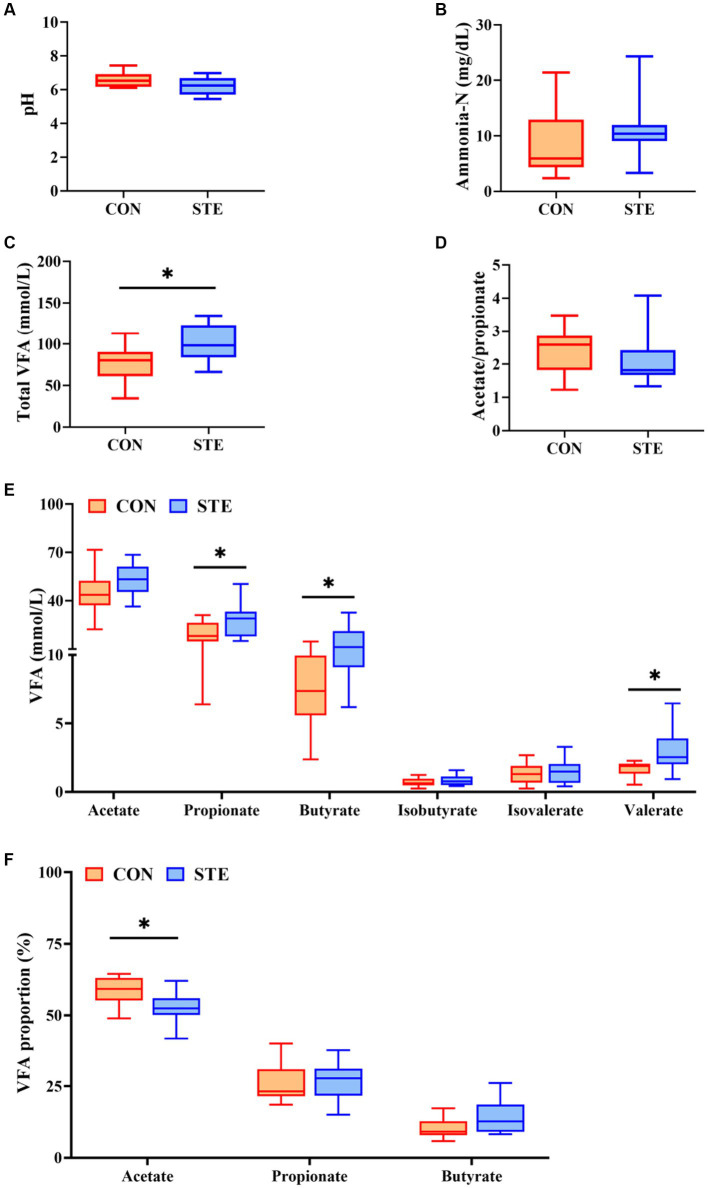
Effects of STE on ruminal fermentation parameters of weaned calves. **(A)** Ruminal pH. **(B)** Ruminal ammonia-N. **(C)** Ruminal total VFA, total VFA = acetate + propionate + butyrate + isobutyrate + isovalerate+ valerate. **(D)** The ratio of ruminal acetate to propionate. **(E)** The levels of ruminal VFAs. **(F)** The proportions of ruminal acetate, propionate and butyrate in Total VFA. Data were presented as the minimum to maximum (*n* = 12 per group). ^*^p < 0.05 compared to the CON group.

As shown in Figures 3A,B,D, ruminal pH (*p* = 0.073), ammonia-N concentration (*p* = 0.268), and the ratio of acetate to propionate (*p* = 0.269) showed no significant changes between CON and STE groups, while ruminal pH in STE group showed a downward trend (*p* = 0.073). In addition, the concentration of total VFA (*p* = 0.012) in the STE group was significantly higher than that in the CON group (Figure 3C). Compared with the CON group, the ruminal concentrations of propionate (*p* = 0.038), butyrate (*p* = 0.013) and valerate (*p* = 0.021) increased in STE group. In contrast, the concentrations of acetate (*p* = 0.102), isobutyrate (*p* = 0.306), and isovalerate (*p* = 0.691) remained unchanged (Figure 3E). The proportion of acetate in the STE group was lower than in the CON group (*p* = 0.013). In contrast, the proportions of propionate (*p* = 0.549) and butyrate (*p* = 0.056) had no significant change (Figure 3F), and the proportion of butyrate (*p* = 0.056) showed an increasing trend.

### 3.4 Effects of STE treatment on the rumen bacterial community richness and diversity in weaned calves

**Figure 4:**
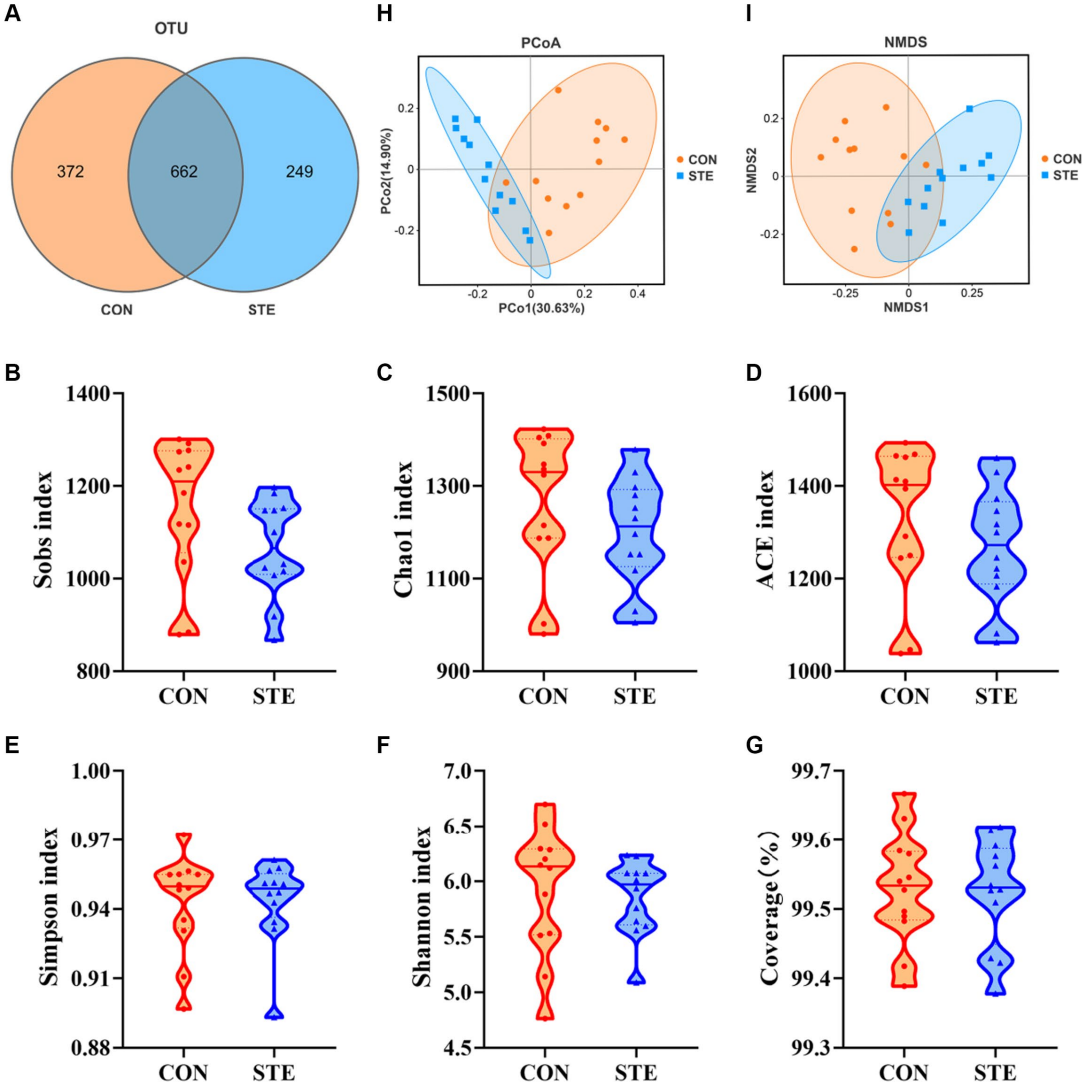
Effects of STE treatment on the rumen bacterial community richness and diversity in weaned calves. **(A)** OTU analysis. Alpha-diversity indexes **(B)** Sobs, **(C)** Chao1, **(D)** ACE, **(E)** Simpson, and **(F)** Shannon indexes in CON and STE group. The sequencing saturation **(G)** Coverage. Data were presented as the minimum to maximum. Welch’s test identified the difference between the two groups (*n* = 12 per group). Beta-diversity analysis **(H)** PCoA plot and **(I)** NMDS plot based on the weighted UniFrac metrics (*n* = 12 per group) of rumen microbiota among CON and STE groups.

To explore how the supplementation of STE altered ruminal fermentation, DNA was extracted from ruminal digesta, and 16S rRNA gene sequencing was performed to study the changes in ruminal microbiota. A total of 8,588 OTUs were identified in the rumen by microbial 16S rDNA profiling. The Venn diagram also revealed that two groups shared 662 common OTUs, while 372 and 249 unique OTUs were illustrated in CON and STE groups, respectively (Figure 4A). The sequencing saturation coverage of both groups of samples was greater than 99% presented in Figure 4G, reflecting the diversity of bacterial community types and structures in the rumen. As observed in Figures 4B–F, the community richness (Sobs, Chao1, and ACE indexes) and diversity (Simpson and Shannon indexes) of rumen bacteria showed no significant changes after adding STE to the diet of calves (*p* > 0.05). PCoA and NMDS (Stress = 0.086) of the OTUs based on weighted UniFrac metrics showed a ruminal microbial community of the STE group was distinct from the CON group (Figures 4H,I).

### 3.5 Effects of STE treatment on the rumen bacterial community at the phylum and genus levels in weaned calves

**Figure 5:**
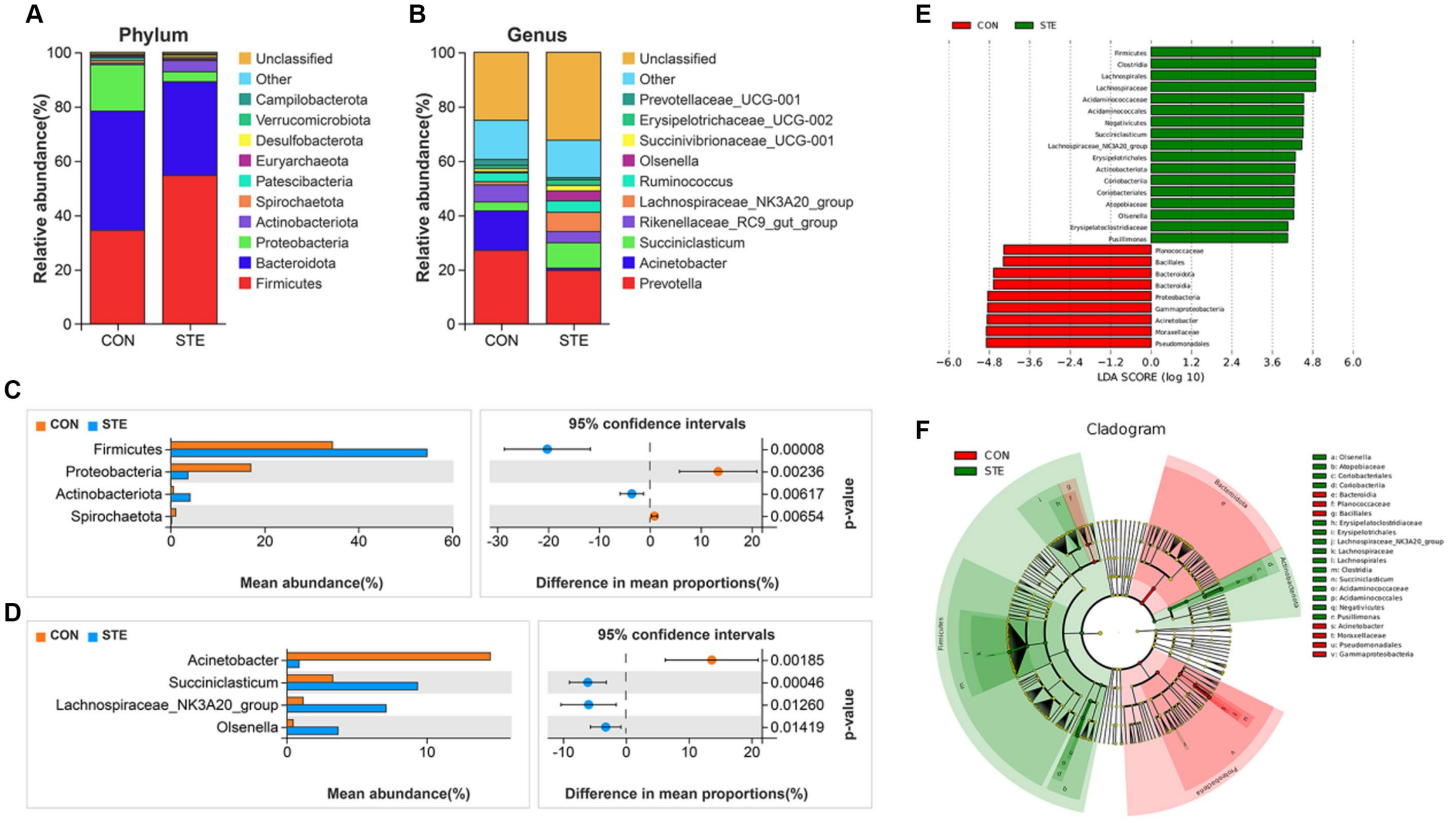
Effects of STE treatment on the rumen bacterial community at the phylum and genus levels in weaned calves. The top 10 relatively abundant bacteria at **(A)** the phylum level (Supplementary Table S1) and **(B)** the genus level (Supplementary Table S2) in the rumen digesta of weaned calves. The differential bacteria at the **(C)** phylum and **(D)** genus levels in the rumen digesta. Welch’s test identified the difference between the two groups. **(E)** LDA score. The LDA score was derived from the LEfSe analysis, which showed that the biomarker taxa LDA score >4 of rumen microbiota in CON and STE groups. **(F)** Cladogram of CON and STE group. A cladogram showed the relationships among taxa at phylum, class, order, family, and genus levels was generated according to LEfSe analysis (*n* = 12 per group).

The top 10 most abundant bacteria at the phyla level of the ruminal digesta are presented in Figure 5A. The dominant phyla were *Firmicutes* (CON vs. STE, 34.48% vs. 54.65%) and *Bacteroidota* (CON vs. STE, 43.88% vs. 34.52%) in the rumen bacterial samples, followed by *Proteobacteria* (CON vs. STE, 17.07% vs. 3.65%), *Actinobacteriota* (CON vs. STE, 0.54% vs. 4.10%), *Spirochaetota* (CON vs. STE, 1.07% vs. 0.18%). The relative abundance of microbial phyla and genera was compared between CON and STE groups to assess the changes in the rumen bacterial communities. At the phylum level, the relative abundances of *Firmicutes* (CON vs. STE, 34.48% vs. 54.65%, *p* < 0.001) and *Actinobacteriota* (CON vs. STE, 0.54% vs. 4.10%, *p* = 0.006) were markedly increased, while that of *Proteobacteria* (CON vs. STE, 17.07% vs. 3.65%, *p* = 0.002) and *Spirochaetota* (CON vs. STE, 1.07% vs. 0.18%, *p* = 0.007) were reduced in STE group (Figure 5C).

In addition, a total of 34 genera were identified, with the top 10 genera in relative abundance shown in Figure 5B. The main dominant genera were *Prevotella* (CON vs. STE, 27.09% vs. 19.71%), *Acinetobacter* (CON vs. STE, 14.55% vs. 0.87%), *Succiniclasticum* (CON vs. STE, 3.28% vs. 9.35%), *Rikenellaceae_RC9_gut_group* (CON vs. STE, 6.28% vs. 4.10%) and *Lachnospiraceae_NK3A20_group* (CON vs. STE, 1.16% vs. 7.10%). At the rumen bacteria genus level, STE supplementation significantly increased the relative abundance of *Succiniclasticum* (CON vs. STE, 3.28% vs. 9.35%, *p* < 0.001), *Lachnospiraceae_NK3A20_group* (CON vs. STE, 1.16% vs. 7.10%, *p* = 0.013) and *Olsenella* (CON vs. STE, 0.45% vs. 3.67%, *p* = 0.014), whereas it reduced the abundance of *Acinetobacter* (CON vs. STE, 14.55% vs. 0.87%, *p* = 0.002) (Figure 5D).

To identify specific microbial biomarkers in the two groups, the data was analyzed using LEfSe selection (Figure 5E), and a cladogram was generated (Figure 5F) by LEfSe analysis of the rumen microbiota community. The microorganisms with an LDA score > 4 were defined as unique, mainly belonging to the phylum level of *Firmicutes*, *Bacteroidota*, *Proteobacteria*, and *Actinobacteriota*. At the genus level, *Acinetobacter* (*p* = 0.001) was significantly enriched in CON group, *Pusillimonas* (*p* = 0.033), *Lachnospiraceae_NK3A20_group* (*p* = 0.001), *Olsenella* (*p* < 0.001) and *Succiniclasticum* (*p* < 0.001) were significantly enriched in STE group, and these bacteria genera had a significant effect on sample grouping.

### 3.6 Correlation between rumen microbiota and VFA level

**Figure 6:**
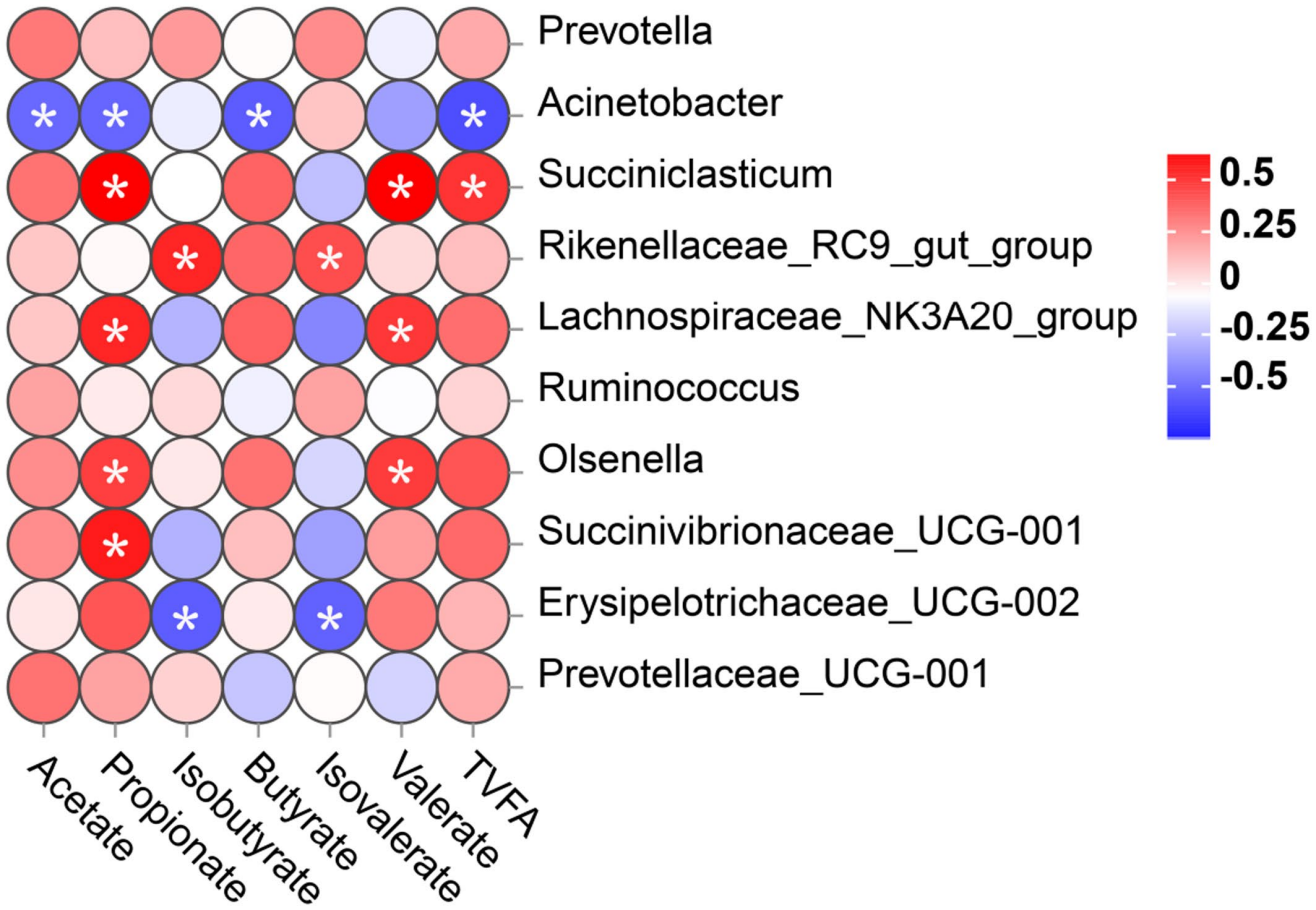
Correlation between rumen bacterial communities (at genus level) and the level of VFA in rumen digesta. Positive correlations (red circles) and negative correlations (blue circles) were illustrated in the heatmap by Spearmen correlation analysis. ^*^p < 0.05 significance for correlation.

To investigate whether different microbial communities in the rumen of calves interact with fermentation parameters, a Spearman correlation was conducted on the correlation between the top 10 bacteria genera and VFA concentration was analyzed, as shown in Figure 6. In this study, the relative abundance of *Acinetobacter* was significantly negatively correlated with the levels of acetate, propionate, butyrate, and TVFA. The relative abundance of *Erysipelotrichaceae_UCG-002* was significantly negatively correlated with isobutyrate and isovalerate levels. However, *Succiniclasticum*, *Lachnospiraceae_NK3A20_group*, *Olsenella*, and *Succinivibrionaceae_UCG-001* showed a significant positive relationship with the propionate level. *Succiniclasticum*, *Lachnospiraceae_NK3A20_group*, and *Olsenella* displayed a significant positive correlation with the level of valerate. In addition, the abundance of *Rikenellaceae_RC9_gut_group* was significantly positively correlated with the concentrations of isobutyrate and isovalerate. The abundance of *Succiniclasticum* was positively correlated with the concentration of TVFA.

## 4 Discussion

The rumen is the first stomach of ruminants, and the level of rumen development directly affects the subsequent production performance of ruminants. With the introduction of pellets at weaning and the subsequent establishment of the rumen microflora, the rumen not only develops physically but also begins to perform metabolic functions. The physical development of the rumen encompasses two key aspects. Firstly, the growth of rumen weight and capacity, and secondly, the development of rumen papillae (Liang et al., 2019). Rumen quality is stimulated by the quantity and physical structure of the ration consumed by the animal (Zitnan et al., 1998). Increased early intake of solid feed pellets stimulates increased rumen mass and growth of rumen teats. In our study, STE supplementation significantly increased rumen weight. It has been reported that the increase in rumen weight is due to the physical stimulation of solid feed, and the growth and keratinization of rumen papillae are due to chemical stimulation by fermentation products such as VFA (Baldwin and Connor, 2017).

Rumen development parameters (such as nipple length and width) are fundamental indicators of feed digestibility, rumen development, and feed efficiency (Na and Guan, 2022). The length and surface area of the ventral rumen papilla of calves supplemented with STE were increased, indicating that the calves in the STE group may have a larger surface area for absorbing nutrients in the ventral rumen. Previously, a study reported greater papillae development in the rumen’s ventral sac (O’Shea et al., 2016). Researchers suggested that this specific development allows for enhanced absorption of VFA compared to the dorsal sac due to increased contact between the ruminal fluid and the ventral wall. Furthermore, another study demonstrated that butyrate concentration is the most effective activator for promoting rumen papillae growth (Shen et al., 2004; Gorka et al., 2011; Kato et al., 2011). It has been shown that solid feed intake during weaning reduces the gene expressions of Insulin-like growth factor binding proteins 2 (IGFBP 2), IGFBP3, and IGFBP6, causing rumen epithelial cell proliferation in weaned calves to stimulate the growth of rumen papillae (Nishihara et al., 2019). Whether the addition of STE works through this pathway needs further proof. We hypothesized that STE induced calves to consume many pellets after weaning, which enhanced rumen development, increased the weight of the forestomach, stimulated nipple growth, promoted muscle development, and enhanced absorption capacity.

In order to promote the normal development of the rumen epithelium, the rumen must have a normal microbial flora to establish viable fermentation, and VFA in the rumen can promote the normal development of the papilla (Bannink et al., 2016). Therefore, we performed an analysis of rumen fermentation parameters. In this study, rumen pH was not significantly altered and remained within the normal range, supplementing with STE, indicating normal rumen fermentation function and no acidosis in calves. However, the trend of a decrease in pH after supplementing STE may be attributed to an increased intake of calves’ pellets, resulting in higher VFA production. As reported, when the microbial production rate of VFA surpasses the rate at which the rumen epithelium absorbs VFA, it leads to an accumulation of VFA in the rumen, ultimately resulting in a decrease in rumen pH value (Zhang et al., 2018).

As the main fermentation product of rumen microorganisms, a high level of proton release accompanies VFA production, and the absorption of VFA in the rumen epithelium is closely linked to the transport of protons or anions to adjust the pH in the rumen, which can directly affect the activity of microorganisms (Gabel et al., 2002). Bicarbonate-dependent transporters on the apical and basolateral membranes facilitate the exchange of free VFA with bicarbonate (HCO_3_^−^), transferring HCO_3_^−^ into the rumen lumen to increase or maintain luminal pH (Dengler et al., 2014). Undissociated VFA passively diffuses through the epithelial cell membrane, and the released protons are exported to the extracellular space via the Na/H exchanger, regulating the intracellular pH (Zhang et al., 2020). Supplementing 1.0% stevia stalk has been reported to more effectively enhance microbial fermentation activity in the rumen and promote the digestion and absorption of fiber (Zhang et al., 2023). The levels of propionate, butyrate, and TVFA in the STE supplementation group increased significantly in our experiment. Acetate and propionate, absorbed by the rumen epithelium, serve as energy sources or precursors for FA synthesis and gluconeogenesis. On the other hand, around 95% of the absorbed butyrate is metabolized within the rumen epithelium to acetoacetate and D-3-hydroxybutyrate, which act as energy sources for ruminants (Gabel et al., 2002). After weaning, VFA such as butyrate are used as the primary energy source and ketogenic precursor of rumen epithelial cells (Baldwin and Jesse, 1992).

STE has been suggested to stimulate rumen development in calves, possibly due to enhanced energy production. In the rumen, VFA production is related to the total amount of organic matter fermented by rumen microorganisms (Ungerfeld, 2020), while the balance between production and absorption regulates rumen VFA concentrations (Islam et al., 2021). VFA production in the developing rumen begins with the ingestion of solid feed, which provides the substrate for anaerobic fermentation (Kim et al., 2016). Steady increases in solids intake corresponded to increases in rumen VFA concentrations, reaching adult-like concentrations at a very young age (Cristobal-Carballo et al., 2021). This also suggested that the effect of STE on rumen VFA may also be due to the calves’ increased intake of pellets. Artificial sweeteners increased the concentration of propionic acid in mouse feces, and enhanced glycan degradation pathways were observed through metagenomic sequencing, in which glycans are fermented to form various compounds, including VFA, further suggesting a potential bacterium for sweeteners metabolism (Suez et al., 2014). This may explain the apparent variation in VFA observed in the STE group in this experiment. Since the metabolic pathways used to synthesize VFA are encoded in the genome of the rumen-content microbiota or the rumen epithelial microbiota, further research is needed to confirm the influence of STE on microbiota function.

The calves supplied with STE had higher valerate concentration in their rumen digesta than the CON group. Valerate in rumen digesta was a product of rumen fermentation, synthesized by propionyl-CoA and acetyl-CoA (Van Soest et al., 1991). In the presence of isobutyrate and isovalerate, valerate fulfills the necessary straight carbon chain requirement for cellulolytic rumen bacteria such as *Bacteroides succinogenes* (Bryant and Doetsch, 1954; Zimmermann et al., 2021). Interestingly, rats ingested stevia (rebaudioside A) showed elevated concentrations of cecal valerate and a strong positive correlation between valeric acid concentration and body weight was observed (Nettleton et al., 2019). Appropriate rumen ammonia-N concentration is a prerequisite for rumen microbial protein synthesis, which is conducive to the growth and reproduction of cellulolytic bacteria. Previous research found that the optimal concentration of ammonia-N for microbial protein synthesis in the rumen should be greater than 5 mg/100 mL (Karimizadeh et al., 2017). The concentration of ammonia-N in this experiment was at a normal level, indicating normal fermentation of rumen microorganisms. However, Zhang et al. (2023) found that the concentration of ammonia-N increased for 48 h, which is inconsistent with the results of this study. This observation may be attributed to two main factors: *in vitro* fermentation and feed type.

The growth and development of calves are primarily related to the rumen microbial community (Malmuthuge and Guan, 2017). Gastrointestinal microbial diversity is crucial in maintaining a stable microbial community. The alpha diversity analysis indicated that the difference between the two groups was non-significant. Research on beef cattle has indicated that rumens with higher microbial diversity can ferment a broader range of substrates, resulting in lower feed conversion rates. Conversely, rumen with less diversity, known as “simple rumen,” may generate more specific products that the host can efficiently absorb and utilize (Li et al., 2019). Therefore, the role of rumen microorganisms may depend on their heritability or function rather than their richness and diversity.

The increased abundance of *Firmicutes* is associated with higher ADG in animals and is vital in influencing feed efficiency (Min et al., 2019). There is limited research on the effects of STE on the microbiome of livestock and poultry, especially about the ruminants we describe. The abundance of *Firmicutes* exhibited a significant increase in the rumen of the STE group, and studies have shown that they can degrade fiber and cellulose (Michalak et al., 2021). Vamanu et al. (2019) used an *in vitro* simulated intestinal method and found that sodium saccharin significantly reduced the abundance of *Firmicutes*, mainly due to the increased abundance of *E. coli* caused by sodium saccharin, which lowered intestinal pH and disrupted the original microbial community. This may be due to the different metabolic characteristics of artificial sweeteners and natural sweeteners in animal bodies and differences in the pathways affecting microorganisms. It has been reported that STE positively affected the intestinal flora of broiler chickens, increasing the concentration of *Streptococcus* and reducing the concentration of *E. coli* in the cecum (Wu et al., 2019). The residue of stevia has been shown to have a significant inhibitory effect on the growth of harmful bacteria while promoting the proliferation of beneficial bacteria (Xiao et al., 2022). However, we did not find that supplementing with STE in the diet significantly inhibited the abundance of harmful bacteria such as *Escherichia coli* in the rumen.

In our study, the relative abundance of *Succiniclasticum*, *Lachnospiraceae_NK3A20_group*, and *Olsenella* was raised in the rumen of calves in the STE group. *Succiniclasticum* predominantly relies on starch and various other carbohydrates as their primary source of fermentation substrates. They metabolize these substrates through a series of biochemical reactions, yielding propionate. Moreover, *Succiniclasticum* can ferment fiber and cellulose (An et al., 2005). Ma et al. (2022) found that *Succiniclassicum* was significantly enriched in the foregut of Aohan fine-wooled sheep and was involved in the metabolism of succinate in LEfSe analysis. At the same time, He et al. (2021) observed that *Succinniclassicum* can utilize succinate to produce propionate, which is beneficial for intestinal health. *Succiniclasticum* significantly increased in the STE group, indicating that carbohydrates in the substrate are beneficial for the reproduction of succinate solubilizing bacteria (Wang et al., 2022). *Lachnospiraceae_NK3A20_group* is an important constituent of the gastrointestinal microbiota in ruminants, characterized by cellulose decomposition activity and starch hydrolysis, which can produce acetate and formate and is closely associated with butyrate production (Russell and Rychlik, 2001). *Lachnospiraceae_NK3A20_group* was positively correlated with the levels of TVFA, propionate, butyrate, isobutyrate, and isovalerate, whereas negatively correlated with pH, acetate concentration, acetate-to-propionate ratio, and xylanase activity (Liu et al., 2022). This is consistent with this study’s positive correlation between *Lachnospiraceae_NK3A20_group* and propionate concentration.

*Lachnospiraceae* can degrade fibrous substances in the intestine to generate VFA, lower pH values, prevent harmful microbial reproduction, and maintain intestinal microbiota homeostasis. A notable finding was that the abundance of *Lachnospiraceae_NK3A20_group* was significantly higher in the rumen of calves in the STE group. This increase in abundance is believed to promote rumen development through enhanced production of butyrate, which directly impacts the development of the rumen. As the abundance of *Lachnospiraceae_NK3A20_group* increased, calves’ rumen utilization rate increased significantly, enabling calves to obtain more energy while promoting rumen growth and development. Zhao (2023) found that artificial sweetener neotame significantly increased the abundance of *Lachnospiraceae NK3A20_group* in the rumen. This phenomenon can be the reason for the increase of the rumen butyrate. This is consistent with our results.

*Olsenellae* is the primary bacterium responsible for choline degradation in healthy and acidotic bovine rumen. Petri et al. (2013) reported that *Olsenella* dominates during subacute rumen acidosis in beef cattle. There is a significant negative correlation between *Olsenella* and propionate concentration in the rumen of Holstein cows (Guo et al., 2023). On the contrary, it was shown that the abundance of *Olsenella* in the rumen of yaks fed with high-concentrate diets was significantly lower than that of yaks fed with low-concentrate diets (Khafipour et al., 2009). The relative abundance of *Olsenella* in the rumen of fattened cattle was negatively correlated with the methane phenotype, positively correlated with the percentage of propionate, and negatively correlated with the acetate-to-propionate ratio (Smith et al., 2022). This may prove that the relative abundance of *Olsenella* increased by supplementing STE, which may also reduce methane production and positively correlate with VFA production in the rumen.

Interestingly, *Olsenella* is a lactobacillus that ferments carbohydrates into lactate (Khafipour et al., 2009). It has been found in the gastrointestinal tract of humans and animals to ferment starch and glycogen substrates, producing lactate, acetate, and formate (Goker et al., 2010). However, the abundance of *Acinetobacter* in the rumen was significantly lower in the STE-supplemented rumen in this experiment. *Acinetobacter* is a Gram-negative aerobic bacterium. Most *Acinetobacter* infections occur primarily in immunocompromised hosts. Usually, *Acinetobacter* infections are characterized by localized suppurative inflammation; in severe cases, meningitis and sepsis may occur (Chebotar et al., 2014). Meanwhile, *Acinetobacter* was shown to have a higher relative abundance in the rumen epithelium of goats in the high-concentrate diet group compared to the low-concentrate group (Shen et al., 2019). So far, most known *Acinetobacter* genus members exhibit pathogenic and commensal lifestyles in the gastrointestinal tract (Shen et al., 2019). Therefore, STE supplementation may have a positive effect on rumen health.

In addition, valerate level was significantly negatively correlated with the abundance of rumen bacterial genera *Olsenella* (Li et al., 2022), which is inconsistent with the results of this experiment. Isobutyrate and isovalerate levels are significantly correlated with the abundance of rumen bacterial genera *Rikenellaceae_RC9_gut_group* (Li et al., 2022), which is consistent with the results of this experiment. Interestingly, we found that the abundance of *Succiniclassicum* increased after supplementing with STE and was positively correlated with the propionate concentration. More importantly, *Succinivibrionaceae_UCG-001* and *Rikenellaceae_RC9_gut_group* were positively correlated with rumen weight (Li et al., 2022). This may prove our results that STE promotes rumen weight, which is related to the microorganisms in the rumen, and the good fermentation of rumen microorganisms produces metabolites that promote rumen development. Thus, changes in rumen microbial abundance correlate with observed phenotypic changes in fermentation parameters, and further studies are needed to examine, alter, and optimize microbial populations to improve rumen homeostasis and promote rumen development. We speculate that the appropriate STE concentration can promote the rumen’s microbial fermentation activity and improve the rumen digestive function of calves, thereby improving feed digestibility, promoting rumen development, and maintaining body health.

## 5 Conclusion

In summary, we found that the rumen weight, papilla length, and papilla surface area of calves supplemented with STE significantly increased compared to the CON group. In addition, calves fed STE displayed increased propionate, butyrate, and TVFA levels in the rumen. STE treatment impacted the rumen microbiota composition in weaned calves. The relative abundance of *Firmicutes* and *Actinobacteriota* in the rumen of calves fed with STE significantly increased, while those of *Proteobacteria* and *Spirochaetota* significantly decreased at the phylum level. At the genus level, *Succiniclasticum*, *Lachnospiraceae_NK3A20_group*, and *Olsenella* significantly increased, while the relative abundance of *Acinetobacter* significantly reduced. This also indicates that it may be possible to enhance fermentation efficiency by modifying the composition of bacterial communities to promote rumen development in weaned calves. Therefore, STE may be a beneficial feed additive to enhance weaned calves’ rumen development.

## Data availability statement

The raw sequence data reported in this paper have been deposited in the Genome Sequence Archive (Genomics, Proteomics & Bioinformatics 2021) in National Genomics Data Center (Nucleic Acids Res 2022), China National Center for Bioinformation/Beijing Institute of Genomics, Chinese Academy of Sciences (GSA: CRA016842) that are publicly accessible at https://ngdc.cncb.ac.cn/gsa.

## Ethics statement

The animal study was approved by the experiment was conducted at the experimental farm of Yangzhou University (Yangzhou, China), and the Yangzhou University Institution Animal Care and Use Committee (IACUC) approved all procedures involving animals (SYXK (Su) 2021-0026). The study was conducted in accordance with the local legislation and institutional requirements.

## Author contributions

KW: Conceptualization, Data curation, Formal analysis, Methodology, Software, Visualization, Writing – original draft, Writing – review & editing. MJ: Methodology, Validation, Writing – review & editing. YC: Investigation, Visualization, Writing – review & editing. YH: Investigation, Methodology, Visualization, Writing – review & editing. ZC: Validation, Writing – review & editing. OD: Writing – review & editing. SJ: Validation, Writing – review & editing. LZ: Resources, Writing – review & editing. YL: Resources, Writing – review & editing. GZ: Funding acquisition, Project administration, Writing – review & editing. ML: Funding acquisition, Project administration, Writing – review & editing.
