# Supplementary material for: Changes in the rumen development, rumen fermentation, and rumen microbiota community in weaned calves during steviol glycosides treatment
Source: Front Microbiol. 2024 Jun 24;15:1395665. doi: 10.3389/fmicb.2024.1395665 (PMC11228177; doi:10.3389/fmicb.2024.1395665)
Supplement: Supplementary file 1 [file Table_1.docx]

Supplementary Table 1. Effects of STE on relative abundance at phylum level of weaned calves (%)

| Phylum | CON | STE |
| --- | --- | --- |
| *Firmicutes* | 34.48 | 54.65 |
| *Bacteroidota* | 43.88 | 34.52 |
| *Proteobacteria* | 17.07 | 3.65 |
| *Actinobacteriota* | 0.54 | 4.10 |
| *Spirochaetota* | 1.07 | 0.18 |
| *Patescibacteria* | 0.88 | 0.36 |
| *Euryarchaeota* | 0.49 | 0.67 |
| *Desulfobacterota* | 0.40 | 0.75 |
| *Verrucomicrobiota* | 0.08 | 0.15 |
| *Campilobacterota* | 0.09 | 0.10 |

Supplementary Table 2. Effects of STE on relative abundance at genus level of weaned calves (%)

| Genus | CON | STE |
| --- | --- | --- |
| *Prevotella* | 27.09 | 19.71 |
| *Acinetobacter* | 14.55 | 0.87 |
| *Succiniclasticum* | 3.28 | 9.35 |
| *Rikenellaceae_RC9_gut_group* | 6.28 | 4.1 |
| *Lachnospiraceae_NK3A20_group* | 1.16 | 7.1 |
| *Ruminococcus* | 3.23 | 4.19 |
| *Olsenella* | 0.45 | 3.67 |
| *Succinivibrionaceae_UCG-001* | 1.17 | 2.06 |
| *Erysipelotrichaceae_UCG-002* | 1.16 | 2.03 |
| *Prevotellaceae_UCG-001* | 2.16 | 0.81 |
